# Genes Required for Survival in Microgravity Revealed by Genome-Wide Yeast Deletion Collections Cultured during Spaceflight

**DOI:** 10.1155/2015/976458

**Published:** 2015-01-13

**Authors:** Corey Nislow, Anna Y. Lee, Patricia L. Allen, Guri Giaever, Andrew Smith, Marinella Gebbia, Louis S. Stodieck, Jeffrey S. Hammond, Holly H. Birdsall, Timothy G. Hammond

**Affiliations:** ^1^Faculty of Pharmaceutical Sciences, The University of British Columbia, Vancouver, BC, Canada V6T 1Z3; ^2^Donnelly CCBR, University of Toronto, Toronto, ON, Canada M5S 3E1; ^3^Durham VA Medical Center, Research & Development Service, Durham, NC 27705, USA; ^4^Bioserve Space Technologies, University of Colorado, Boulder, CO 80309, USA; ^5^The Institute for Medical Research, Durham, NC 27705, USA; ^6^Department of Veterans Affairs Office of Research and Development, Washington, DC 20420, USA; ^7^Departments of Otorhinolaryngology, Immunology, and Psychiatry, Baylor College of Medicine, Houston, TX 77030, USA; ^8^Nephrology Division, Department of Internal Medicine, Duke University School of Medicine, Durham, NC 27705, USA; ^9^Nephrology Section, Department of Internal Medicine, George Washington University School of Medicine, Washington, DC 20052, USA

## Abstract

Spaceflight is a unique environment with profound effects on biological systems including tissue redistribution and musculoskeletal stresses. However, the more subtle biological effects of spaceflight on cells and organisms are difficult to measure in a systematic, unbiased manner. Here we test the utility of the molecularly barcoded yeast deletion collection to provide a quantitative assessment of the effects of microgravity on a model organism. We developed robust hardware to screen, in parallel, the complete collection of ~4800 homozygous and ~5900 heterozygous (including ~1100 single-copy deletions of essential genes) yeast deletion strains, each carrying unique DNA that acts as strain identifiers. We compared strain fitness for the homozygous and heterozygous yeast deletion collections grown in spaceflight and ground, as well as plus and minus hyperosmolar sodium chloride, providing a second additive stressor. The genome-wide sensitivity profiles obtained from these treatments were then queried for their similarity to a compendium of drugs whose effects on the yeast collection have been previously reported. We found that the effects of spaceflight have high concordance with the effects of DNA-damaging agents and changes in redox state, suggesting mechanisms by which spaceflight may negatively affect cell fitness.

## 1. Introduction

Physical effects of microgravity during spaceflight can often be described by equations that allow their quantification [1, 2]. For example, microgravity has well-defined effects on sedimentation in association with reduced terminal velocity and shear in suspension culture [3] and reduced gravity-dependent convection of gases [4]. Biological effects of spaceflight on cells and organisms, on the other hand, are much harder to define [1, 2]. For example, spaceflight also entails radiation exposure, which has been studied in diverse systems but whose effects are not fully understood [5]. What is needed is a robust, unbiased, quantifiable system that is relevant for translation to ground-based applications and that is able to clearly distinguish spaceflight effects. It is our premise that yeast deletion collections are ideally suited for this type of analysis as yeast can be precisely controlled genetically and readily grown under spaceflight conditions. Biological responses of yeast strains during spaceflight can be quantified and compared to well-established databases of ground-based stressors and the comparisons can reveal features that are unique to microgravity as well as features that are shared with ground-based perturbations. While yeast cannot completely reflect the complexities of mammalian cells organized into tissues, the high degree of homology shared with human (~70% of all essential yeast genes have a significant human homolog) provides hypotheses for the mechanism of many responses of interest [6, 7].

Previous studies have attempted to identify, isolate, and offset the various physical factors changing during spaceflight to demonstrate their effects in an iterative fashion [8–10]. Earlier studies on the effects of space radiation on yeast failed to find any change in point mutation rates, DNA replication and/or repair, heritable damage, or colony morphology [5, 8, 11, 12]. However, those studies were limited by assay sensitivity. Here we applied the yeast deletion collection as a biological reporter to understand the metabolic pathways affecting survival during culture in spaceflight. In this fashion, we are able to make genome-wide comparisons and test for concordance against an extensive library of more than 3200 physical and pharmacological stressors [13].

Yeast is the first, and to date only, organism for which a complete, genome-wide knockdown collection is available. This collection is comprised of a genome-wide set of strains where each strain carries a precise deletion of a single gene [14]. Assembled over a four-year period by a consortium of 35 laboratories, this collection has been used by hundreds of laboratories to test thousands of different environmental stressors to define the genes required for survival in those conditions (see [15] for review). The molecular barcodes present in each strain allow the yeast deletion collection to be grown as a pool in the presence or absence of the stressor of interest after which the relative abundance of each strain is subsequently quantified [16]. Strains carrying a deletion of a gene required for survival in the presence of the stressor grow more slowly and thus exhibit a fitness defect reflected by their reduced abundance at the end of the culture period. In this manner, all genes required for growth can be readily identified in a single experiment, revealing the genes and associated pathways affected by the stressor.

To identify the metabolic and genomic pathways affected by spaceflight, the homozygous and heterozygous yeast deletion collections were grown in spaceflight and ground control conditions, with and without hyperosmolar sodium chloride, providing a second stressor. In spaceflight alone, the homozygous deletion collection revealed the importance of processes linked to mitochondria, while the heterozygous collection highlighted genes involved in regulating translation and ribosomal RNA transport. Both homozygous and heterozygous collections highlighted DNA repair. With the addition of NaCl, the homozygous collection also revealed the importance of RNA-related processes including ribosome assembly and biogenesis and mRNA processing and decay, as well as modification of tRNAs. Moreover, the NaCl addition highlighted replication processes more clearly (compared to the homozygous collection without NaCl), suggesting that spaceflight has measurable effects on these core and evolutionarily conserved processes. With the heterozygous collection, the addition of NaCl led to the identification of a nuclear pore organization gene, potentially providing additional insight into how RNA transport is affected by spaceflight. Taken together, the deletion collections identified several biological processes associated with spaceflight, and the additional hyperosmolar stress emphasized the importance of related processes.

In a follow-up analysis, we queried the effects of spaceflight against a database of drug effects on yeast to search for those that are most concordant, thereby suggesting similar mechanisms of perturbation. Not only do the effects of spaceflight have relatively high concordance with the effects of DNA-damaging agents, but also there is tight agreement amongst multiple therapeutic agents in this drug class, providing additional support for these findings.

## 2. Materials and Methods

### 2.1. Overall Design

The Opticell Processing Module, described below, was used to perform a series of ~21 generation pooled growth experiments on two yeast deletion collections: (i) ~4800 homozygous strains and (ii) ~5900 heterozygous strains (including ~1100 single-copy deletions of essential genes), each carrying unique DNA barcodes that act as strain identifiers. Experiments were performed in both rich media and rich media supplemented with 0.5 M NaCl to assess the additional effect of osmotic stress on survival. The samples flew sortie on space shuttle mission STS-135 to the International Space Station (ISS). Parallel control experiments were performed in static 1G terrestrial controls in the Orbital Environmental Simulator at Kennedy Space Center to match temperature, humidity, air composition, and volatile organic compounds. Ground controls were conducted in a 24-hour asynchronous fashion to allow matching of the experimental timelines on ISS as relayed through air-to-ground communication by the flight crew. At the end of the growth period, the fitness of each strain in each experimental pool was assessed as described [17]. Briefly, genomic DNA was extracted from each sample, the barcodes in each pool were amplified by PCR, and the abundance of each barcode was quantified by next generation sequencing. A barcode count reflects the abundance of the corresponding strain at the end of the experiment, that is, a quantification of the relative requirement of the deleted gene for growth in the tested condition. In total, the experiment results in a count for each gene resulting in a gene list rank ordered by their importance for growth in the tested condition.

### 2.2. Yeast Deletion Pool Construction

The yeast deletion collections were stored as individual strains in YPD containing 7% DMSO at −80°C, in 96-well plates. The plates were thawed, mixed, and robotically pinned onto YPD agar plates as an array of 384 strains. After two days of growth at 30°C, colonies were consolidated (four plates of 384 to one plate of 1536 colonies) and robotically pinned in triplicate. Cells were grown in 30°C for 2-3 days until colonies formed. Slow growing strains were grown separately for 2-3 additional days. All plates were then flooded with 5–7 mL of media, scraped and pooled in YPD + 7% DMSO to a final concentration of OD_600_ = 0.84, and frozen at −80°C until use, as described [17].

### 2.3. Construction of Opticell Culture System and Spaceflight Experiment

**Figure 1:**
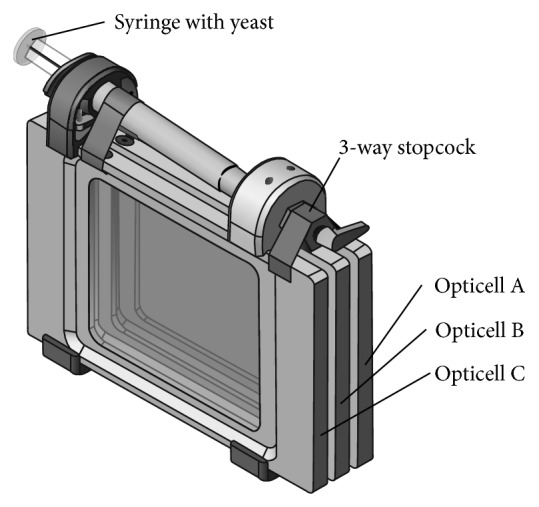
The Opticell Processing Module (OPM) designed for propagation of each deletion collection for ~21 generations of growth. The OPM comprises three commercially available optically clear chambers (Opticells, Nunc) that are joined by a manifold and scaffold that can be autoclaved and assembled rapidly. The manifold contains a multiway valve unit which mates to each Opticell or to an the off position using O-ring seals. The opposite side of the valve contains a Luer fitting into which a standard 3cc syringe is attached. To perform a growth assay in the OPM, each of the three chambers is filled with 7 mL of sterile growth media. Deletion pools are loaded into the inoculation syringe and then injected into Chamber A of the OPM, precooled to 4°C. Growth is initiated by warming the unit to 30°C. After 16–24 h, 0.5 mL is removed from Chamber A and injected and mixed into Chamber B using the same syringe. This is repeated to continue multigenerational growth in Chamber C.

In this study, we designed the Opticell Processing Module or OPM (Figure 1) that was capable of maintaining the yeast deletion collection as a pool grown in liquid culture for at least 20 generations in microgravity. The hardware comprised a liquid-sealed system of growth chambers (Opticells) that allowed for gas exchange across polystyrene membranes. Each OPM consisted of three Nunc Opticells held together with a common manifold and valve system that is autoclaved and attached with watertight O-ring seals. A 3 mL syringe connected to the manifold with a Luer fitting is used to transfer liquid between chambers and mix without breaking sterility and with minimal operator intervention. The valve on the manifold has four settings that connect the syringe to the following port locations 1: Off position, 2: Opticell A, 3: Opticell B, or 4: Opticell C. The OPM allows propagation of each deletion collection for a combined ~21 generations of growth when three chambers are used and the inoculum and transfer volumes are 0.5 mL.

To perform a growth assay in the OPM, each of the three chambers was prefilled with 7 mL of sterile growth media. Deletion collection aliquots were preloaded into each syringe and shipped to Kennedy Space Center, frozen in media containing 7% DMSO (v/v) as a cryoprotectant. During final integration at Kennedy Space Center, the OPMs were prechilled to 4°C. Each deletion collection aliquot was thawed, attached to an OPM manifold, injected, and mixed into chamber A. Cultures were maintained at 4°C and flown to the International Space Station (ISS). The growth experiment was initiated on orbit by warming the OPMs to 30°C. After 16–24 h at 30°C, a 0.5 mL sample was removed from chamber A using the same syringe and inoculated into chamber B. The process was repeated 16–24 hours later to inoculate 0.5 mL of sample from chamber B into chamber C. After an additional 16–24 hours, the OPMs were cooled back down to 4°C to greatly reduce any further growth and preserve the samples for return to Earth and postflight analysis. Exponential yeast growth leads to early depletion of growth media nutrients and significant retardation of further growth well before 16–24 hours. Growth is limited by media volume and strain distribution within the yeast deletion library reaches a steady state within that Opticell.

### 2.4. Next Generation Sequencing

The flight samples that returned from the ISS were handled in parallel with the ground control set. The OPM was disassembled into its three Opticells, and the entire contents were transferred to a storage tube using a blunt needle connected to a 20 mL syringe. One mL of each sample (at a final OD_600_ of 1.0-2.0) was processed to extract genomic DNA. Purified deletion pool DNA was amplified in two separate PCR reactions as described [17] and the amplicons purified prior to sequencing on an Illumina HiSeq2000. Each purified amplicon library was sequenced to a minimum depth of 500 counts/strain/sample as described [18]. Duplicate experiments were performed for all conditions, to ensure that at least one complete time course was collected for each pool (heterozygote and homozygote) and each condition. Due to failures in sample processing, several time points were not recovered or did not meet our in-house quality metrics (e.g., if sequence counts/strain were below threshold values). Accordingly, we focused on evaluating each experimental condition using singleton data as described in Table 1.

### 2.5. Data Analysis

All computational analyses were performed using *R* [19] unless otherwise indicated.

#### 2.5.1. Normalization of Sequence Counts

Sequence counts for each strain in each experiment were quantified and normalized according to [18]. Briefly, each 20-mer barcode was amplified with primers comprised of the common barcode primers in addition to the sequences required for cluster formation on the Illumina flow cell. For multiplexed Illumina sequencing, 5-mer tag sequences were incorporated into each primer between the Illumina and barcode primer sequences. This multiplexing tag allowed postsequencing assignment of each amplicon to a particular experiment. Results for the 7-generation time point of the heterozygous deletion pool grown in spaceflight without NaCl did not pass our quality control and, consequently, that time point was omitted from analyses of the heterozygous pool without NaCl. All other counts were mean-normalized between experiments such that each experiment had the same mean count. We added ten pseudocounts to all sequence tag tallies (and, thus, all subsequent gene tallies) to prevent division by zero during data analyses (see Table S1 and Table S2 for mean normalized counts in Supplementary Materials available online at http://dx.doi.org/10.1155/2014/976458).

#### 2.5.2. Barcode Selection for Each Strain

For each strain, we used signal from only the upstream or the downstream barcode (relative to the deletion site). First, we assumed that barcode counts close to zero represent background noise (e.g., possibly due to incorrect mapping of reads to barcodes). We thus selected a background threshold (*bgThreshold *= 100; see Parameter Selection), assuming that counts below it do not accurately reflect strain abundance. Then, for each time course, we filtered out barcodes where the average (normalized) count for the first 14 generations (the earliest time point with usable data in all experiments) was below* bgThreshold*. This filtering removed all barcodes for ~650–3200 strains (depending on the time course), and these strains were omitted from subsequent analyses; see Supplementary Tables S3 (homozygous strains) and S4 (heterozygous strains).

For time courses that only had two time points (and thus an inefficient number to compute fits), strains that still had two barcodes after filtering were represented by their upstream barcodes due to their overall better behavior observed in a previous study [20]. For other time courses, linear fits (with and without the time logged) were computed for each remaining barcode. We defined the best fit as the fit with the lowest residual sum of squares (RSS) and used the *F*-test to compute a *P* value estimating the significance with which the fitted model is better than the null model (of a flat line at the average count value). The Benjamini and Hochberg method was used to correct the *P* for multiple comparisons and generate FDR values [21]. Strains with two remaining barcodes were represented by the barcode with the higher *R* ^2^ (a measure of the amount of variation in the data explained by the fitted model), because they manifest less noise and better fit the data.

#### 2.5.3. Parameter Selection

The selected normalization method (tested mean and quartile normalization) and* bgThreshold *(tested 50, 100, and 150) is the combination that resulted in the most significant enrichment of slow growing strains identified in the heterozygous deletion pool, sampled every two generations for 20 generations (data not shown), with slow growers identified in a previous study [22]. Briefly, we defined slow growers as those exhibiting sizable decreases in abundance over time. The significance of the decrease was estimated with FDR values (see Barcode Selection for Each Strain), and the magnitude was estimated with ΔAUC = (*〈*area under the growth curve*〉* − *〈*area under the flat growth curve*〉*)/(*t*_max⁡_ − *t*_0_), where the flat growth curve is fixed at the *t*_0_ abundance level, and the area under a curve is estimated using the trapezoid method. Also, if at some time point the abundance of a strain is less than or equal to* bgThreshold* and remains at negligible levels for the rest of the time course, we identified the strain as slow growing.

#### 2.5.4. Identification of Significant Fitness Defects in Time Point Comparisons

To identify strains that exhibited significant fitness defects at a later time point (14 or 21 generations) compared to the first time point (7 generations), normalized counts less than* bgThreshold* were first forced to equal* bgThreshold*. Then, for each strain, we computed log_2_ratio = log_2_(abundance_7G_/abundance_14G/21G_) where abundance_*y*G_ is the count of the strain at *y* generations. For a given time point, robust *Z* scores were computed from the set of log_2_ratios; for example, *Z*_*i*_ = (log⁡_2_ratio_*i*_ − 〈log⁡_2_ratio  median〉)/〈log⁡_2_ratio  MAD〉 for strain *i*. Each *Z*_*i*_ was then used to obtain *P*_*i*_ from the standard normal distribution, and we assume that strains with low *P* values are outliers in the distribution of log_2_ratios. Moreover, strains with counts above* bgThreshold *at the first time point and counts equivalent to* bgThreshold* at the later time point of interest are defined as having dropped out. Taken together, we define strains with significant fitness defects at a specific time point as strains with log_2_ratio ≥ 1 and *P* ≤ 0.001 and/or strains that dropped out (Table S5, Table S6).

#### 2.5.5. Spaceflight versus Ground Comparisons

For comparisons involving specific time points, we identified the set of strains that exhibited significant fitness defects (relative to the first time point) in the flight condition but not in the ground condition. This set is then further restricted to the set of strains with useable data in both conditions.

#### 2.5.6. Gene Ontology (GO) Enrichment Analysis

We obtained gene ontology (GO) annotations of yeast genes from the* Saccharomyces* Genome Database (downloaded on May 26, 2012). GO biological processes that were too specific (containing less than five genes) or too general (containing greater than 300 genes) were excluded from the analysis.

Given a query set of genes (e.g., genes deleted from a set of (flight-ground) strains), we used the hypergeometric test to obtain a *P* value estimating the significance with which the set is enriched with genes annotated to a given biological process, relative to a gene universe defined as the set of genes with usable data for both flight and ground conditions. Due to a limited number of significantly enriched processes following correction for multiple comparisons (FDR ≤ 0.1), here we report significantly enriched processes prior to the correction (*P* ≤ 0.01).

**Figure 2:**
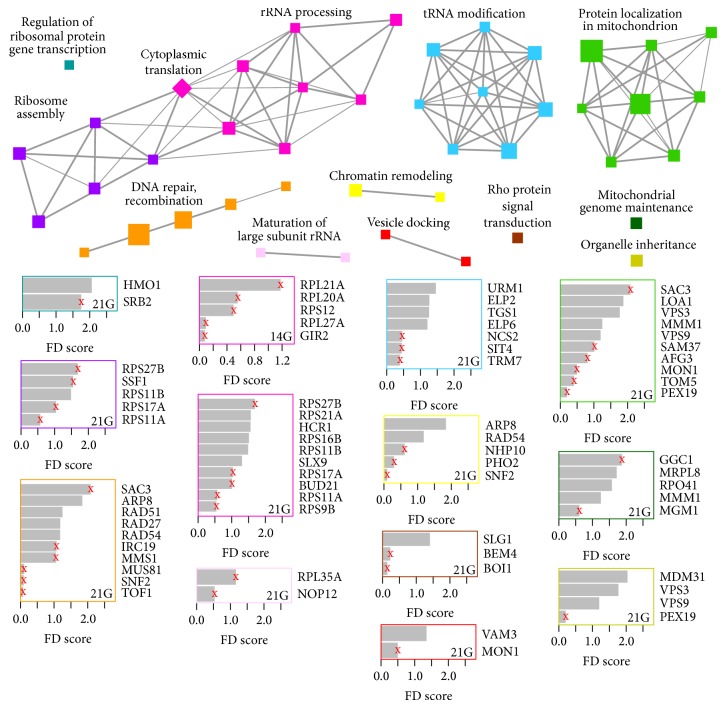
Biological processes enriched amongst genes associated with flight-specific fitness defects at different time points in the homozygous deletion series. Each node represents a significantly enriched gene ontology (GO) biological process (hypergeometric test *P* ≤ 0.01). A circle node indicates enrichment at 14 generations compared to 7 generations (the first time point), a square node indicates enrichment at 21 generations compared to 7 generations, and a diamond node indicates enrichment at both 14 generations and 21 generations (see Methods). Node size is proportional to the significance of enrichment [−log_10_(*P*)]. Node color indicates processes that share genes (see Methods) and summary labels are shown for nodes of the same color. Edges indicate ≥ 50% gene overlap between connected processes; width is proportional to the degree of overlap. Each bar plot provides fitness defect (FD) scores for genes that contribute to the enrichment of processes with the same node color as the plot border. Specifically, the length of a bar is proportional to the log_2_(abundance_7G_/abundance_14G/21G_), where abundance_*y*G_ represents the abundance of the corresponding gene deletion strain at *y* generations (see Methods). An “*x*” on the bar indicates that the abundance of the strain lowers to background level at later time point.

**Figure 3:**
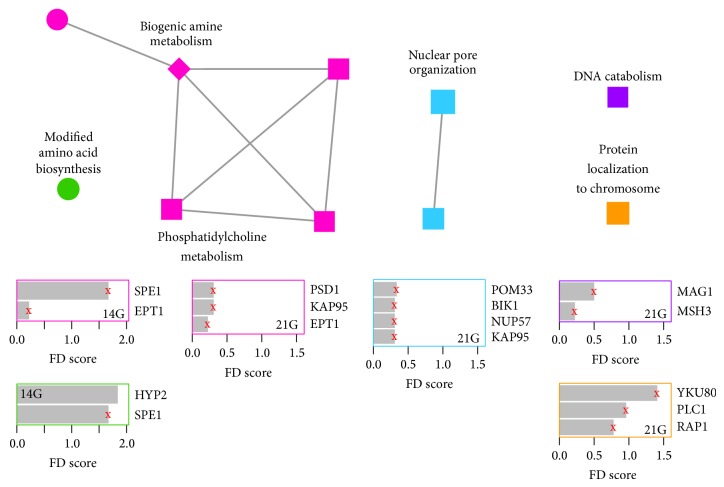
Biological processes enriched amongst genes associated with flight-specific fitness defects in the presence of NaCl, at different time points in heterozygous deletion samples. Each node represents a significantly enriched gene ontology (GO) biological process (hypergeometric test *P* ≤ 0.01). Nodes, edges, and plots are as specified for Figure 2.

We visualized GO enrichment results with enrichment maps shown in Figures 2 and 3 that were generated using an approach similar to the Enrichment Map Cytoscape Plugin v1.1 [23, 24]. In contrast to the plugin, the nodes in each map were clustered with MCL (inflation = 2), using the overlap coefficient computed by the plugin as the similarity metric (coefficients less than 0.5 were set to zero). Nodes in the same cluster were assigned the same node color, and a cluster label was determined based on common themes in the processes within the cluster. Moreover, the size of a node was made to be proportional to the significance with which the corresponding process is enriched [−log_10_(*P*)]. Each bar plot summarizes the genes that contribute the most to the enrichment of processes with the same node color as the plot border. Specifically, a plot shows the flight-associated genes that are annotated to the largest number of relevant processes (if more than 10 genes, only the top 10 are shown). For each gene, the bar length is proportional to a fitness defect measure (i.e., log_2_ratio).

The enrichment maps also combine two sets of enrichment results, with the processes enriched in one set shown with circle nodes, the processes enriched in the second set shown with square nodes, and the processes enriched in both sets shown with diamond nodes.

#### 2.5.7. Similarity between Flight-Associated Genes and Compound-Associated Genes

We previously treated pools of yeast deletion strains with ~3200 compounds separately [13]. Each compound was subsequently associated with a set of genes deleted from strains that exhibited significant fitness defects induced by the compound. Like sets of flight-associated genes in this study, sets of compound-associated genes were assessed for enrichment of genes annotated to specific biological processes (as described above), resulting in an “enrichment profile” for each condition of interest. In each profile, each process is associated with a *P* measuring the significance of enrichment. Similarity between a pair of enrichment profiles was computed by concordance of −log_10_(*P*) across all processes common to both profiles, where concordance is like Pearson correlation except that scale is not ignored. Compounds with enrichment profiles that are most similar to a given flight enrichment profile may induce cellular responses that are most similar to the response induced by flight.

## 3. Results and Discussion

Because we cannot distinguish the individual parameters that include flight, lack of gravity, and increased radiation, for the purposes of this paper, these are referred to collectively as “spaceflight” throughout the text. To measure the effects of spaceflight on the rate of yeast growth in the Opticell, we inoculated 0.5 mL of a yeast deletion pool into 7 mL of YPD, resulting in a starting OD_600_ of ~0.06/mL and incubated at 30°C. Following growth for ~24 hr (~7 generations), 0.5 mL of the saturated culture was inoculated into the second chamber. This process was repeated for the final growth phase in the third chamber. Population doubling time was ~100 min in microgravity compared to ~90 min in ground-based controls. Each sample was grown for seven generations/Opticell for a total of 21 generations (Table 1). Doubling times were back-calculated using the OD_600_ of the samples collected at each time point.

The morphology of Opticell-grown yeast in spaceflight was indistinguishable from static controls when observed by light microscopy; for example, budding pattern, overall shape, and size were not detectably different in the two conditions. On scanning electron microscopy, there were budding polarity and ruffling changes in every field, but there were no consistent differences (data not shown).

We assessed the yeast deletion collection samples for patterns of strain sensitivity in the following manner: barcode counts for each strain in each sample were measured and normalized as described in Methods. The counts were used to rank each strain in each sample in order of their importance for growth. Four different samples were available from both spaceflight and ground cultures: (1) homozygous deletion collection in YPD, (2) homozygous deletion collection in YPD plus 0.5 M NaCl, (3) heterozygous deletion collection in YPD, and (4) heterozygous deletion collection in YPD plus 0.5 M NaCl. Each culture was sampled at three different time points, 7 generations, 14 generations, and 21 generations, and shown in Table 1. Samples from ground controls were compared to the corresponding samples grown in microgravity on the ISS.

We analyzed changes in strain abundance by comparing each time point to a later time point. Using this approach allowed us to capture those strains that became depleted in any seven-generation interval. Strains with sizable decreases in measured abundance or with abundances that drop to background levels (and remain there) were identified as exhibiting fitness defects (FDs). Moreover, strains with flight-specific FDs were identified by subtracting the strains with FDs in the ground condition.

For the purposes of our gene ontology (GO; http://amigo.geneontology.org) enrichment analysis, we considered the homozygous and heterozygous data separately. Based on a wealth of published data [14, 15], the homozygous, nonessential deletion collection tends to reveal a similar set of genes involved in pathways required for resistance to/survival in multiple environmental conditions, whereas the heterozygous collection of all strains tends to be more specific, identifying essential proteins uniquely required for growth in a specific condition [13].

For the homozygous deletion collection, strains that were depleted from the pool specifically in spaceflight conditions are significantly enriched for genes in biological processes related to different aspects of RNA metabolism and catabolism, including ribosome biogenesis, regulation of ribosomal protein transcription, cytoplasmic RNA translation, rRNA processing, tRNA modification, and mRNA decay (Table 2, Figure 2, and Table S5). We also found that processes related to DNA integrity were required for survival in spaceflight. In particular, the linked processes of DNA repair and DNA recombination and replication as well as chromatin remodeling were all required for resistance to the effects of spaceflight. Finally, these DNA repair requirements extend to the mitochondria, which, by virtue of its small genome, is hypersensitive to DNA damage. Consistent with this, we found that genes required for both mitochondrial maintenance and proper protein localization to the mitochondria were enriched in the homozygous samples.

The enrichment of these particular processes is consistent with a general induction of DNA damage, which, in turn, perturbs RNA biogenesis [25]. Interestingly we have previously observed this phenomenon with a class of therapeutics that act as nucleotide analogs, such as 5-fluorouridine and fluorocytosine (described in detail below). Additionally, it is particularly noteworthy that, although we do see evidence of a requirement for RNA and DNA processing genes in spaceflight alone, the requirement is exacerbated when spaceflight is combined with the additional hyperosmotic stress imposed by the addition of 0.5 M NaCl (Table S6). We speculate that the added salt stress potentiates the DNA-damaging effects of spaceflight via the induction of reactive oxygen species (ROS). The ability of salt stress to induce ROS and subsequent DNA damage has been previously reported [26] and, in particular, the yeast mitochondria appears to be hypersensitive to this type of stress, consistent with its small genome being susceptible to the effects of DNA damage [27]. Furthermore, mitochondrial protein abundance has been shown to rapidly increase upon osmotic shock, and therefore the enrichment for mitochondrial protein localization we observe may reflect this requirement.

To gain further insight into the pathways that modulate the response to both microgravity stress and combined spaceflight and salt stress, we used the GO enrichment profiles to query a database of over 3200 distinct drug treatments of the yeast deletion collections [13]. Specifically we quantified the similarity between the GO enrichments by computing the concordance of −log_10_(*P*) between any two profiles, where *P* measures the significance of enrichment of a single GO category. These concordance values are similar to Pearson correlation values; that is, values closer to one indicate greater similarity between profiles, except that high concordance also requires the scale of values to be similar between the profiles. When calculating concordance, we focused on GO biological process enrichment profiles (Table S7).

One of the strong concordances was observed with 5-fluorouridine (0.42), an FDA-approved anticancer drug that is thought to also act by two mechanisms: (i) inhibiting thymidylate synthetase and (ii) through metabolism into cytotoxic ribonucleotides and deoxyribonucleotides that can be incorporated into DNA and RNA (Table 3) [14]. In addition to being incorporated in DNA and RNA, we and others have shown that the drug has been shown to inhibit the essential ribonuclease activity of the exosome complex [28]. Similarly, carmofur, a derivative of 5-fluorouracil, displays a concordance of 0.34. A similar concordance is seen with 5-fluorocytosine (5-FC), whose activity is identical to 5-fluorouracil (5-FU). Finally, 8-methoxypsoralen, a DNA-damaging agent that, upon photoactivation, conjugates and forms covalent bonds with DNA, shows a congruence of 0.32. This compound causes the formation of both monofunctional (addition to a single strand of DNA) and bifunctional adducts (crosslinking of psoralen to both strands of DNA) that ultimately result in cell death.

We also found high concordance to the diallyl disulfide profile (0.40), an agent that has been demonstrated to be efficient for detoxification of a variety of cells. Diallyl disulfide and related garlic derivatives have been shown to significantly increase the production of the enzyme glutathione S-transferase (GST), which binds electrophilic toxins in the cell. Overloading the cell with inhibitory doses of diallyl disulfide reveals genes required for survival in the presence of increased reactive oxygen species (ROS) [29].

In the case of the heterozygous collection, we found significant GO enrichments for the following categories: lipid metabolism, DNA catabolism, and regulation of translation and posttranslational modification (specifically protein phosphorylation) (Figure 3). As expected (based on previous studies of the heterozygous collection), both the number of genes associated with FDs and the number of enriched categories are considerably smaller than those derived from the homozygous collection [30]. This likely reflects two related phenomena: first, genes that when deleted in heterozygotes are sensitive to spaceflight encode proteins that participate in the pathways identified in the homozygous collection, where the fitness defect is stronger because the gene is completely absent. Second, none of these heterozygote strains encode a direct target of the perturbation.

Interestingly, when we searched for drug profiles with high concordance with the spaceflight profiles derived from the heterozygous collection, we detected modest concordance with two human chemotherapeutics, mitoxantrone (concordance = 0.19), and Epirubicin (congruence 0.142). Both of these agents damage DNA by intercalating into the DNA double helix and also by stabilizing the cleavable complex that is the substrate of topoisomerase II [31–33].

## 4. Conclusions

The experiments presented here represent a proof of principle for conducting full genome environmental screens in spaceflight using robust hardware that can recapitulate a full automation suite with environmental control in the space of a small suitcase. The performance of this platform is significant for spaceflight studies and promises to enable terrestrial experiments in extreme environments that will have direct application to microbial bioprocessing for manufacturing, alternative fuel development, and basic research. The results from these experiments suggest that spaceflight has subtle but significant effects on core cellular processes including growth control via RNA and ribosomal biogenesis, metabolism, modification, and decay pathways. Furthermore, significant roles for DNA repair and replication, response to pH signaling, control of gene expression, and mitochondrial function were observed. The yeast chemogenetic analysis of spaceflight samples presented here strongly implicates DNA and RNA damage as the major ground-based analogs of spaceflight stress. Given the unique and substantial radiation exposure in space, this is consistent with major radiation-mediated effects. Unfortunately a 1 g control on ISS that might have allowed better discrimination between the contributions of space radiation versus the effects of microgravity on yeast responses was not available to us at this time. Current on-going experiments are designed explore these effects and dissect them from other potentially confounding variables. The high concordance to the profile induced by diallyl disulfide suggests increased glutathione S-transferase, binding of electrophilic toxins, increased reactive oxygen species, and change in redox state. These pathways, which are required for survival in spaceflight, can guide future experiments in two fundamental ways: firstly by suggesting environmental modifications that can bolster cellular and organismal integrity by avoiding further stress to these pathways, and secondly, by identifying drug stresses that can exacerbate these pathway requirements in an effort to control pathological cell growth in the case of proliferative diseases.

## Supplementary Material

Supplementary tables 1 and 2 provide the Illumina sequencing counts for each barcode/strain of each sample following mean normalization for the homozygous strains (Supplementary Table S1) and the heterozygous deletion strains (Supplementary Table S2). Supplementary tables 3 and 4 list those strains in the homozygous deletion collection (Supplementary Table S3) and the heterozygous deletion collection (Supplementary Table S4) that were excluded from the analysis for technical reasons. Supplementary tables 5 and 6 contain the results from the analysis of the homozygous deletion collection, comparing ground experiments to flight experiments in YPD media (Supplementary Table S5) and in YPD media + 0.5M NaCl (Supplementary Table S6). Supplementary table S7 contains the concordance in GO enrichments of the experiments presented in this study to a compendium of published data (reference 13). Supplementary tables 8 and 9 contain the results from the analysis of the heterozygous deletion collection, comparing ground experiments to flight experiments in YPD media (Supplementary Table S8) and in YPD media + 0.5M NaCl (Supplementary Table S9).

## Figures and Tables

**Table 1:** Experimental samples collected and available for analysis.

Condition	Zygosity	Generations
Ground	Homozygous	7, 14, 21
Ground + 0.5 M NaCl	Homozygous	7, 14, 21
Flight	Homozygous	7, 14, 21
Flight + 0.5 M NaCl	Homozygous	7, 14, 21
Ground	Heterozygous	14, 21^*^
Ground + 0.5 M NaCl	Heterozygous	7, 14, 21
Flight	Heterozygous	14, 21^*^
Flight + 0.5 M NaCl	Heterozygous	7, 14, 21

^*^7-generation samples from the indicated condition were not available for analysis due to failures in sample processing or failure to meet in-house quality metrics as described in Methods.

**Table 2:** Effects of spaceflight on yeast genome responses identified with the homozygous deletion series.

General pathway	GO biological process
RNA metabolism and catabolism	(i) Ribosome biogenesis (ii) Regulation of ribosomal protein transcription (iii) Cytoplasmic RNA translation (iv) rRNA processing (v) tRNA modification (vi) mRNA decay

DNA integrity	(i) DNA repair (ii) Recombination and replication (iii) Chromatin remodeling (iv) Mitochondrial maintenance (v) Proper protein localization to the mitochondria

**Table 3:** Concordance between drug effects and spaceflight effects on yeast genome responses identified with the homozygous deletion series (+NaCl).

Drug (concordance)	Biological function
5-Fluorouridine (0.42) 5-Fluorouracil (0.36) Carmofur (0.34) 5-Fluorocytosine (0.35)	Pyrimidine analogs that inhibit thymidylate synthase and are metabolized into cytotoxic ribonucleotides and deoxyribonucleotides that can be incorporated into DNA and RNA

8-Methoxypsoralen (0.32)	DNA-damaging agent

Diallyl disulfide (0.4)	Increased glutathione-S-transferase changes redox state by binding electrophilic toxins

## Abbreviations

AUC: Area under the curve
BP: Biologic processes
DMSO: Dimethyl sulfoxide
FD: Fitness defect
FDR: False discovery rate
GO: Gene ontology
GST: Glutathione S-transferase
ISS: International Space Station
MAD: Median absolute deviation
MCL: Markov Clustering Algorithm
OD: Optical density
OPM: Opticell Processing Module
PCR: Polymerase chain reaction
ROS: Reactive oxygen species
RSS: Residual sum of squares
YPD: Yeast peptone dextrose.
