# Supplementary material for: Genes Required for Survival in Microgravity Revealed by Genome-Wide Yeast Deletion Collections Cultured during Spaceflight
Source: Biomed Res Int. 2015 Jan 13;2015:976458. doi: 10.1155/2015/976458 (PMC4309212; doi:10.1155/2015/976458)
Supplement: Supplementary file 1 — Supplementary tables 1 and 2 provide the Illumina sequencing counts for each barcode/strain of each sample following mean normalization for the homozygous strains (Supplementary Table S1) and the heterozygous deletion strains (Supplementary Table S2). Supplementary tables 3 and 4 list those strains in the homozygous deletion collection (Supplementary Table S3) and the heterozygous deletion collection (Supplementary Table S4) that were excluded from the analysis for technical reasons. Supplementary tables 5 and 6 contain the results from the analysis of the homozygous deletion collection, comparing ground experiments to flight experiments in YPD media (Supplementary Table S5) and in YPD media + 0.5M NaCl (Supplementary Table S6). Supplementary table S7 contains the concordance in GO enrichments of the experiments presented in this study to a compendium of published data (reference 13). Supplementary tables 8 and 9 contain the results from the analysis of the heterozygous deletion collection, comparing ground experiments to flight experiments in YPD media (Supplementary Table S8) and in YPD media + 0.5M NaCl (Supplementary Table S9). [file 976458.f1.zip › Supplementary_Tables.docx]

Supplementary Table S1: mean normalized counts, homozygous strains

Supplementary Table S2: mean normalized counts, heterozygous strains

Supplementary Table S3: omitted homozygous deletion strains

Supplementary Table S4: omitted heterozygous deletion strains

Supplementary Table S5: homozygous strain analyzed data, ground-to-flight

Supplementary Table S6: homozygous strain analyzed data, ground-to-flight + 0.5M NaCl

Supplementary Table S7: Concordance in GO enrichments to published data

Supplementary Table S8: heterozygous strain analyzed data, ground-to-flight

Supplementary Table S9: heterozygous strain analyzed data, ground-to-flight + 0.5M NaCl
